# Author’s Reply; Nucleic Acid Testing for the Detection of HBV DNA

**DOI:** 10.5812/kowsar.1735143x.758

**Published:** 2011-10-01

**Authors:** Mohammad Kazemi Arababadi

**Affiliations:** 1 Department of Microbiology, Hematology and Immunology, Faculty of Medicine, Rafsanjan University of Medical Sciences, Rafsanjan, IR Iran; 2 Infectious and Tropical Diseases Research Center, Rafsanjan University of Medical Sciences, Rafsanjan, IR Iran

**Keywords:** Nucleic Acids, Hepatitis B

Dear Editor,

First, we agree with Seto et al. regarding the impacts of the sensitivity of the detection methods of OBI (occult hepatitis B infection) prevalence but disagree regarding their idea on the lack of usefulness of anti-HBc screens. They have referred to Seo et al., who reported that the prevalence of anti-HBc positivity in Korea was 13.5%, whereas the prevalence of OBI was 0.016% [1]. Anti-HBc is the first antibody that is produced against HBV and is the most stable antibody [2]; hence, the presence of the antibody in individuals demonstrates introduction of HBV to the immune system via several mechanisms, including transfusion [3],[4]. Therefore, it seems that the rate of OBI among anti-HBc-positive persons is more than the anti-HBc-negative blood donors [5]. During blood screening programs, hepatitis B surface antigen (HBsAg) testing, followed by polymerase chain reaction (PCR) assays, will be the most accurate approach of identifying OBI, but some blood transfusion services are unable to use PCR (based on high  costs). We have proposed applying anti-HBc as a screening test for blood transfusion services that are unable to use PCR to reduce the risk of PTH via OBI-infected blood and its components.

Second, Seto et al. have presented a nice mechanism for the possible cause for cryptogenic HCC in endemic chronic hepatitis B regions. They have described a good, probable mechanism of undetectable virological HBV markers in tumor histology. These mechanisms should be examined in future studies.
